# Supplementary material for: Antibacterial Potential Analysis of Novel α-Helix Peptides in the Chinese Wolf Spider Lycosa sinensis
Source: Pharmaceutics. 2022 Nov 21;14(11):2540. doi: 10.3390/pharmaceutics14112540 (PMC9698133; doi:10.3390/pharmaceutics14112540)
Supplement: Supplementary file 1 [file pharmaceutics-14-02540-s001.zip › pharmaceutics-2009858-supplementary.pdf]

Supplementary Information

Antibacterial potential analysis of novel α-helix peptides in the Chinese wolf spider *Lycosa sinensis*

Huaxin Tan[1], Junyao Wang[1], Yuxin Song[1], Sisi Liu[1], Ziyan Lu[1], Haodang Luo[3*] and Xing Tang[2*]

1. Department of Biochemistry and Molecular Biology, The Key Laboratory of Ecological Environment and Critical Human Diseases Prevention of Hunan Province Department of Education, School of Basic Medicine, Hengyang Medical School, University of South China, Hengyang, 421001, China
2. Hunan Key Laboratory for Conservation and Utilization of Biological Resources in the Nanyue Mountainous Region, College of Life Sciences, Hengyang Normal University, Hengyang, 421002, China
3. Department of Clinical Laboratory, The Affiliated Nanhua Hospital, Hengyang Medical School, University of South China, Hengyang, 421001, China

Email: haodangluo89@gmail.com (H.L.); xtang2018@hynu.edu.cn (X.T.)

A

Peptide name	Homology AMPs			
	Name	APD ID	Similarity percentage	Organism
LS-AMP-E1	Uperin 2.7	AP00321	45.83%	Toad
LS-AMP-F1	GLK-19	AP00142	45.45%	Synthetic
LS-AMP-G1	Latarcin 3b	AP01013	50.00%	Spider

B

```
LS-AMP-E1      ---AGMKNIIDAIKKKLGGKL----------     18
Uperin 2.7     --------GIIDIAKKLVGGIRNVLGI----     19
LS-AMP-F1      ---TGLGKIGYLMKKLLSKAKV---------     19
GLK-19         -----------GLKKLLGKLLKKLGKLLLK     19
LS-AMP-G1      SWAVMLPDLVRQ--KLKQRW-----------     18
Latarcin 3b    SWASMAKKLKEYMEKLKQRA-----------     20
```

Figure S1. The sequence homology matching by the Antimicrobial Peptide Database (A). Multiple sequence alignment of LS-AMPs with homology AMPs (B).

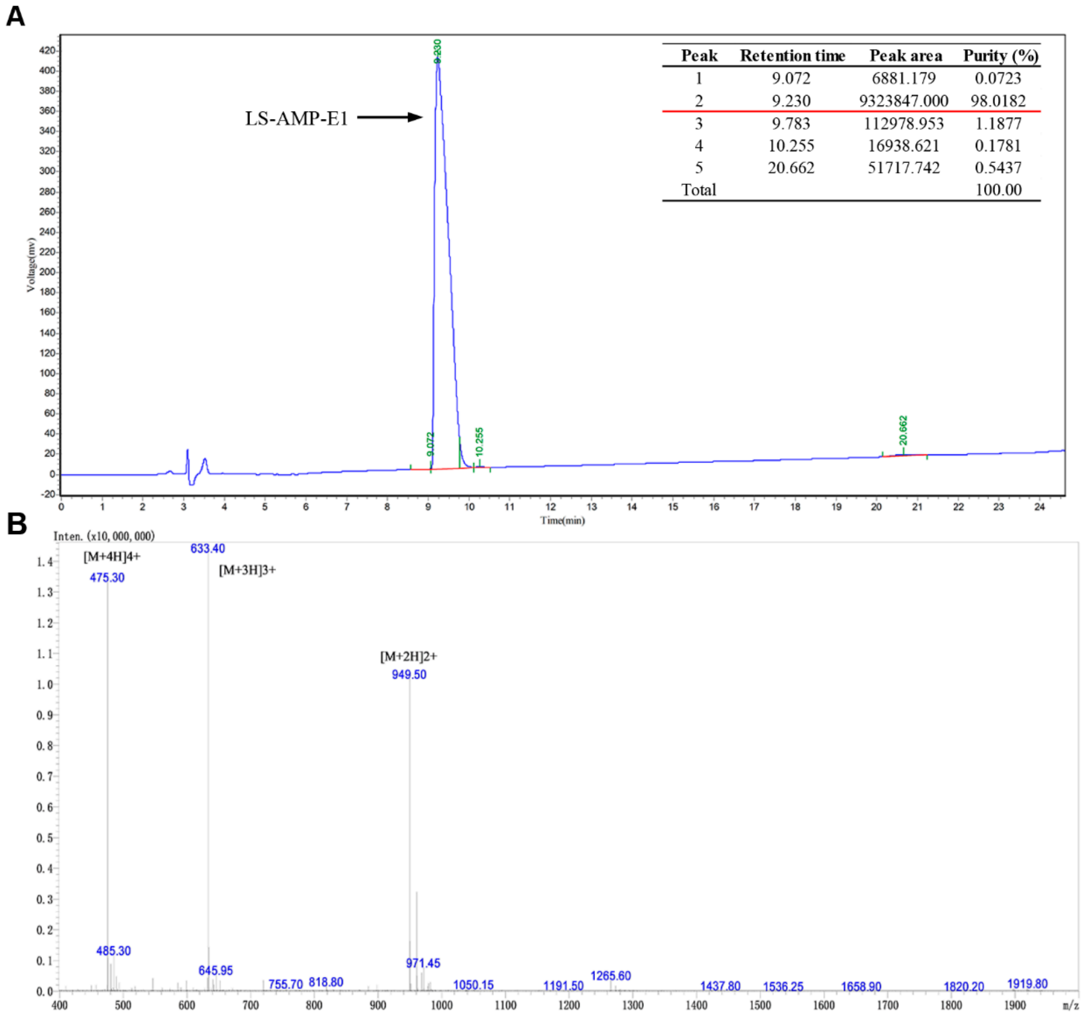

Figure S2. The High-performance liquid chromatography (HPLC) (A) and Mass spectrometry (MS) (B) profiles of LS-AMP-E1.

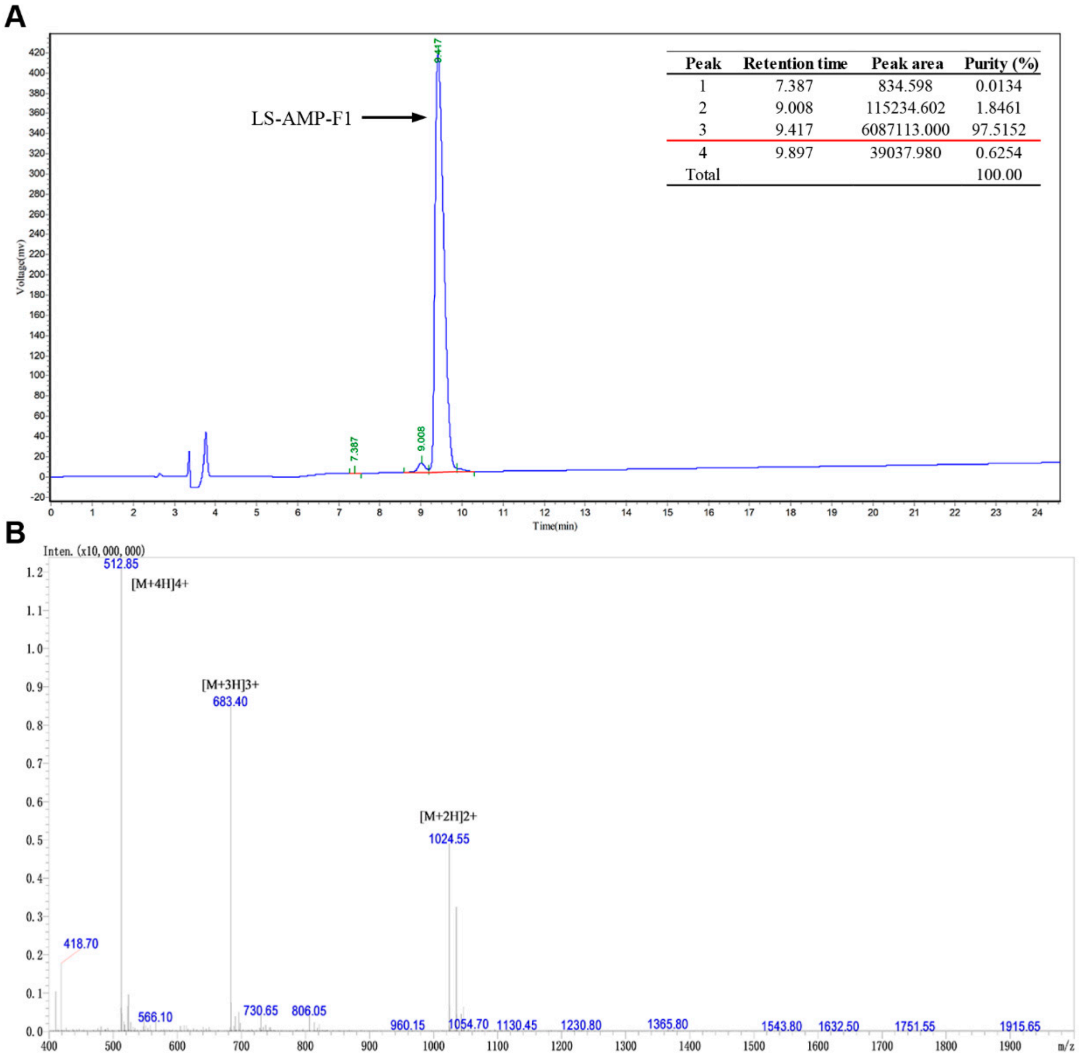

Peak	Retention time	Peak area	Purity (%)
1	7.387	834.598	0.0134
2	9.008	115234.602	1.8461
3	9.417	6087113.000	97.5152
4	9.897	39037.980	0.6254
Total			100.00

Figure S3. The HPLC (A) and MS (B) profiles of LS-AMP-F1.

A

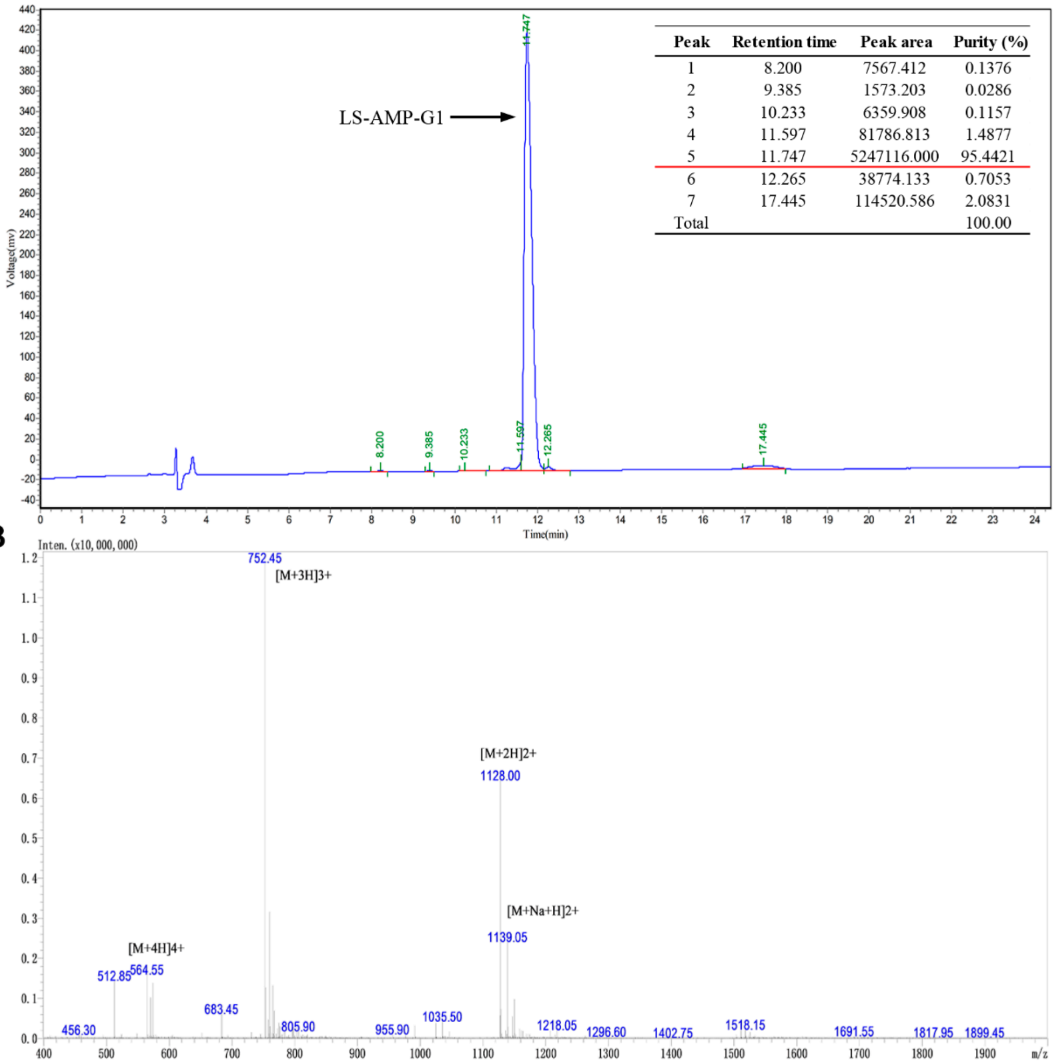

Peak	Retention time	Peak area	Purity (%)
1	8.200	7567.412	0.1376
2	9.385	1573.203	0.0286
3	10.233	6359.908	0.1157
4	11.597	81786.813	1.4877
5	11.747	5247116.000	95.4421
6	12.265	38774.133	0.7053
7	17.445	114520.586	2.0831
Total			100.00

B

Figure S4. The HPLC (A) and MS (B) profiles of LS-AMP-G1.

Table S1. The clinical profiles of MDR strains isolated from 30 cases.

Species	Number	Resistant type	Age range	Disease	Specimen type	Sample number
E. faecium	1359	None	80-90	UTI	urine	20220505117
	1320	VRE	20-30	AP	blood	20220405146
S.aureus	1090	MRSA	40-50	CI	urine	20211101018
	1022	MRSA	60-70	injury	sputum	20210930047
	1065	MRSA	50-60	diabetic foot	excretion	20211020125
	1081	MRSA	70-80	head trauma	sputum	20211027035
K.pneumoniae	1049	CR	40-50	open injuries	excretion	20211017048
	1096	None	60-70	brainstem infarction	blood	20211105003
	1041	CR	0-10	fever	urine	20211013047
	1044	CR	50-60	HE	blood	20211014205
A.baumannii	1055	CR	50-60	buring	excretion	20211018150
	1023	CR	60-70	stroke	sputum	20211005014
	1025	CR	70-80	abdominal pain	sputum	20211008184
	1032	CR	70-80	abdominal pain	urine	20211009174
	1038	CR	70-80	abdominal pain	blood	20211012080
	1040	CR	60-70	gout	BALF	20211013103
P.aeruginosa	1121	CR	50-60	aneurysm	sputum	20211115011
	1088	CR	80-90	pneumonia	sputum	20211031041
	1099	CR	80-90	pneumonia	sputum	20211104085
	1095	CR	60-70	COPD	sputum	20211104106
E.coli	1270	CR	70-80	prostatic hyperplasia	urine	20220223151
	1172	CR	70-80	hematencephalon	urine	20211215062
	1188	CR	70-80	hematencephalon	urine	20211224059
	1064	ESBLs	70-80	notalgia	urine	20211019073
	1010	ESBLs	70-80	pancreatitis	blood	20210915118
	1278	ESBLs	70-80	abdominal pain	excretion	20220301065
	1165	ESBLs	60-70	abdominal pain	urine	20211212047
	1288	ESBLs	50-60	ureteral stones	blood	20220304189
	1170	ESBLs	60-70	hematencephalon	sputum	20211214079
	1156	ESBLs	70-80	UTI	blood	20211206179

VRE: Vancomycin-resistant enterococcus; MRSA: Methicillin-resistant Staphylococcus aureus; CR: Carbapenem-Resistant; ESBLs: extended spectrum beta-lactamases; UTI: urinary tract infection; AP: acute pancreatitis; CI: cerebral infarction; HT: Hepatic Encephalopathy; COPD: chronic obstructive pulmonary disease; BALF: bronchoalveolar lavage fluid.

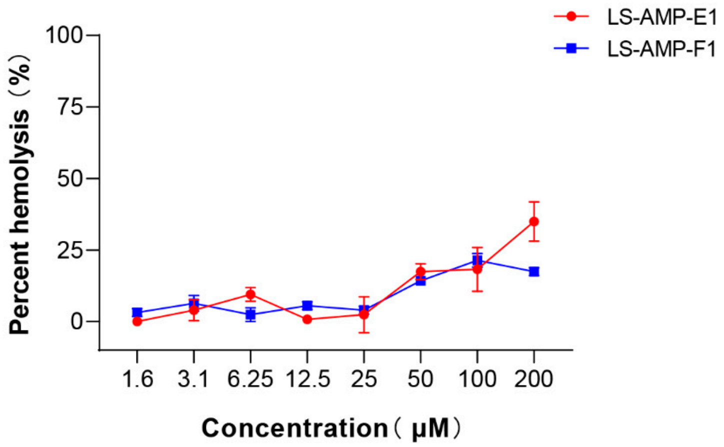

Figure S5. The hemolytic effects of LS-AMP-F1 and LS-AMP-E1 on mouse erythrocytes. Data represent means ± SD of 3 individual experiments.

Table S2. The MICs of the checkerboard test for standard strains.

Peptides/Antibiotics		Strains	
		E. coli CCTCC AB 2018675	*S. albus* CGMCC 1.3374
**MIC LS-AMP-E1 (μM)**	**Alone**	6.25	12.5
	**with AZI**	0.3906	0.3906
**MIC AZI (μg/mL)**	**Alone**	0.05	3.1
	**with LS-AMP-E1**	0.025	0.775
**MIC LS-AMP-E1 (μM)**	**Alone**	6.25	12.5
	**with ERY**	1.5625	0.3906
**MIC ERY (μg/mL)**	**Alone**	6.25	6.25
	**with LS-AMP-E1**	1.5625	1.5625
**MIC LS-AMP-E1 (μM)**	**Alone**	6.25	12.5
	**with DOX**	0.1953	12.5
**MIC DOX (μg/mL)**	**Alone**	1.6	0.025
	**with LS-AMP-E1**	0.4	0.0008
**MIC LS-AMP-F1 (μM)**	**Alone**	12.5	6.25
	**with AZI**	3.125	3.125
**MIC AZI (μg/mL)**	**Alone**	0.05	3.1
	**with LS-AMP-F1**	0.0016	0.003
**MIC LS-AMP-F1 (μM)**	**Alone**	12.5	6.25
	**with ERY**	3.125	0.1953
**MIC ERY (μg/mL)**	**Alone**	6.25	6.25
	**with LS-AMP-F1**	0.0977	1.5625
**MIC LS-AMP-F1 (μM)**	**Alone**	12.5	6.25
	**with DOX**	3.125	1.5625
**MIC DOX (μg/mL)**	**Alone**	1.6	0.025
	**with LS-AMP-F1**	0.2	0.00625
**MIC Lycosin-I (μM)**	**Alone**	3.1	1.6
	**with AZI**	0.775	0.05
**MIC AZI (μg/mL)**	**Alone**	0.05	3.1
	**with Lycosin-I**	0.025	0.775
**MIC Lycosin-I (μM)**	**Alone**	3.1	1.6
	**with ERY**	0.0969	0.05
**MIC ERY (μg/mL)**	**Alone**	6.25	6.25
	**with Lycosin-I**	3.125	1.5625
**MIC Lycosin-I (μM)**	**Alone**	3.1	1.6
	**with DOX**	0.0484	0.025
**MIC DOX (μg/mL)**	**Alone**	1.6	0.025
	**with Lycosin-I**	0.4	0.025

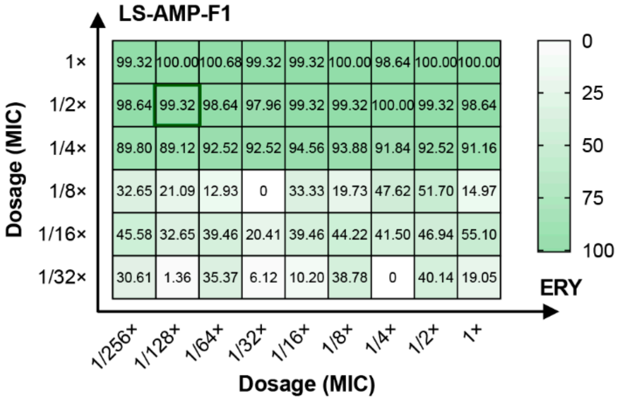

Figure S6. Inhibition rate of the checkerboard test for MDR *E. faecium* (1320).

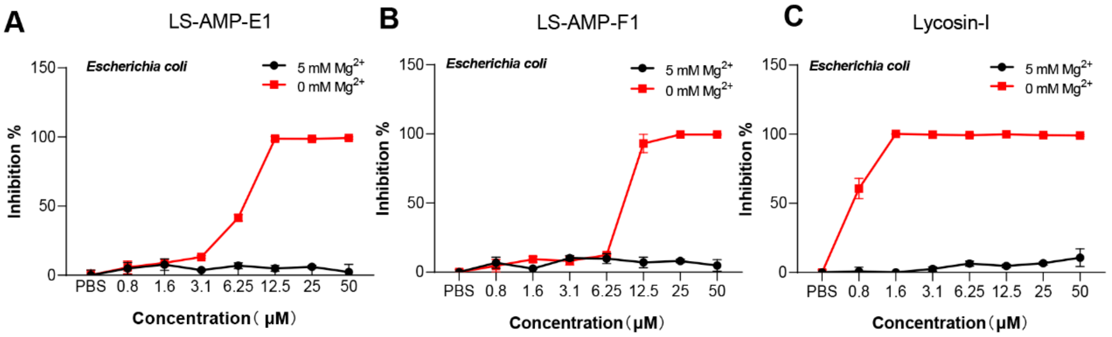

Figure S7. Effect of Mg^{2+} on the antimicrobial activity of LS-AMP-E1 (A), LS-AMP-F1 (B) and Lycosin-I (C) against a strain of *E. coli*.
